# Antioxidant Effects of Carnosine-Enriched Chicken Meat Consumption in Athletes: Modulation of SOD Activity and Superoxide Levels—A Randomized Control Trial

**DOI:** 10.3390/antiox14091069

**Published:** 2025-08-30

**Authors:** Nikolina Kolobarić, Leon Perić, Zrinka Mihaljević, Petar Šušnjara, Alina Boris, Ines Drenjančević, Ivana Jukić, Ana Stupin

**Affiliations:** 1 Department of Physiology and Immunology, Faculty of Medicine Osijek, Josip Juraj Strossmayer University of Osijek, 31000 Osijek, Croatiazmihaljevic@mefos.hr (Z.M.); ines.drenjancevic@mefos.hr (I.D.); 2 Scientific Centre of Excellence for Personalized Health Care, Josip Juraj Strossmayer University of Osijek, 31000 Osijek, Croatia; 3 Department of Ophthalmology, University Hospital Center Osijek, 31000 Osijek, Croatia; 4 Department of Interdisciplinary Sciences, Faculty of Kinesiology Osijek, Josip Juraj Strossmayer University of Osijek, 31000 Osijek, Croatia

**Keywords:** antioxidants, athletes, carnosine, functional food

## Abstract

Dipeptide carnosine has gained attention for its antioxidant and anti-inflammatory effects demonstrated in preclinical studies, but evidence from human trials remains limited. This study investigated whether dietary carnosine delivered through enriched chicken meat can modulate redox status in competitive athletes. This randomized controlled trial involved 35 male competitive athletes who were assigned to either a control group (N = 16; CTRL) consuming regular chicken meat (410 mg/day) or a carnosine group (N = 19; CAR) receiving carnosine-enriched chicken meat (590 mg/day) for 21 days. Blood sample collection, cells isolation and anthropometric measurements were performed before and after the intervention to assess antioxidant enzyme activity, intracellular reactive oxygen species production, 8-iso Prostaglandin F2α (8-iso PGF 2α) concentration, and cell adhesion molecules serum concentrations. Results were expressed as mean ± standard deviation (SD). Group comparisons were conducted using parametric and non-parametric tests, ANCOVA was applied to assess post-intervention differences adjusted for baseline values, while a two-way ANOVA was performed to determine the significance of interactions between time and treatment for each parameter, significance set at *p* < 0.05. CAR group showed a significant reduction in serum 8-iso PGF 2α and increased SOD activity compared to baseline and the CTRL group. Intracellular hydrogen peroxide and peroxynitrite production increased, while superoxide anion production decreased in the CAR group. Carnosine-enriched chicken meat consumption significantly reduced lipid peroxidation, increased serum enzyme activity, and decreased superoxide anion production in competitive athletes. While further research is needed to elucidate the mechanisms and key factors behind it, the observed changes indicate that carnosine-enriched chicken meat consumption affects SOD activity consequently producing an antioxidative effect.

## 1. Introduction

Recently, increasing efforts have been made to develop new strategies aimed at preventive healthcare and improving the quality of life, such as the use of functional foods [1,2]. Functional foods are considered an advanced type of food product that offers certain benefits to human health due to their enrichment with bioactive compounds [3,4,5,6]. The term “nutraceuticals”—a combination of “nutrition” and “pharmaceuticals”—refers to products that resemble regular food but are suggested to possess preventive and therapeutic properties [4,7,8,9,10]. This concept has gained considerable popularity over the past decade, especially among younger individuals seeking to mitigate the effects of ageing and unhealthy lifestyles [11]. However, genetic factors, general dietary habits, and physical activity—which play a significant role in how nutrients are absorbed and utilized in the body—are often overlooked.

Dipeptide carnosine and related compounds have been quoted in the literature as reactive oxygen species (ROS) scavengers and heavy metal chelators, achieving these effects by forming complexes that are less reactive or inhibitory to metal-dependent reactions [10,12,13,14,15]. A large number of studies involving carnosine supplementation/treatment in rodent or cell line models corroborate these claims. It was found that carnosine successfully decreases oxidative stress and ROS levels in human kidney-2 (HK-2), murine microglial (BV-2), macrophage (RAW 264.7), and pre-osteoblastic (MC3T3-E1) cell lines [16,17,18,19]. Furthermore, carnosine increased antioxidant enzyme activity/expression (superoxide dismutase, SOD; glutathione peroxidase, GPx) and decreased nicotinamide adenine dinucleotide phosphate oxidase (NADPH oxidase) expression both in vitro and in vivo [16,17,19,20,21]. Carnosine further upregulated anti-inflammatory interleukin 10 (IL-10) and transforming growth factor beta 1 (TGF-β1), while it downregulated pro-inflammatory tumour necrosis factor alpha (TNFα), IL-1β, and IL-6 in BV-2 and RAW 264.7 cells [17,18]. It was also reported that carnosine increases nuclear factor erythroid 2-related factor 2 (Nrf2) expression in Sprague Dawley (SD) rats, zebrafish and murine cells [19,20], which is significant due to Nrf2’s ability to upregulate antioxidative enzymes such as SOD and heme oxygenase-1 (HO-1), thus protecting against oxidative damage and serving as a potential preventive agent [19,22,23,24,25].

Several randomized controlled studies involving human subjects investigated oral carnosine supplementation effects on blood glucose and fasting insulin in overweight non-diabetic, pre-diabetic, and type 2 diabetes (T2D) adults. It was found that 1–2 g of carnosine per day decreased glucose and insulin levels, even though there were no significant changes detected in monocyte chemoattractant protein-1 (MCP-1), IL-6, TNFα, IL-10, or high-sensitivity C-reactive protein (hsCRP) concentrations [26,27,28,29]. In healthy adults, 2 g of carnosine supplementation per day resulted in improved speed and cognitive efficiency mainly among the younger cohort participants [30], while 4 g of carnosine per day in highly trained athletes attenuated exercise-induced glutathione loss as well as reduced oxidative stress markers [31].

Even though there is a growing and promising evidence of carnosine antioxidant effects in rodent and cell models, there is a lack of clinical trials to determine its true impact on human health. Our research group has recently reported that carnosine supplementation delivered via functional food improves peripheral vascular function by enhancing endothelium-dependent and vascular smooth muscle-dependent vasodilation in both microcirculation and macrocirculation [10], and may therefore serve as a useful tool for the prevention of chronic diseases associated with inflammation. Therefore, in the present study, we aimed to explore the potential of dietary carnosine supplementation through enriched chicken meat to affect oxidative stress status, antioxidant defence, and markers of endothelial activation in competitive athletes by assessing relevant biomarkers.

## 2. Materials and Methods

### 2.1. Study Design and Participants

**Figure 1:**
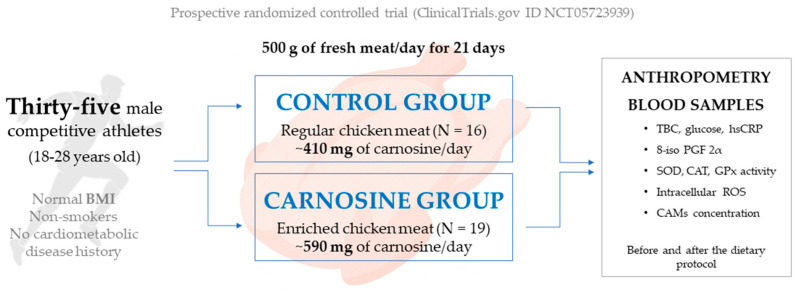
Study design. BMI—body mass index; TBC—total blood count; hsCRP—high sensitivity C-reactive protein; 8-iso PGF 2α—8-iso prostaglandin F2α; SOD—superoxide dismutase; CAT—catalase; GPx—glutathione peroxidase; ROS—reactive oxygen species; CAMs—cell adhesion molecules.

The current study was designed as a prospective randomized controlled trial (ClinicalTrials.gov ID NCT05723939). Thirty-five young, competitive male athletes were recruited from local sports clubs and randomly assigned to one of two experimental groups: (1) Control group (CTRL)—participants consumed regular chicken meat (N = 16); and (2) Carnosine group (CAR)—participants consumed carnosine-enriched chicken meat (N = 19). Inclusion/exclusion criteria involved ages 18 to 45, normal body mass index (BMI), non-smokers, and no history of diabetes, hypertension, hyperlipidaemia, kidney damage, coronary, cerebrovascular, or artery disease. The study protocol lasted 21 days, during which participants consumed 500 g of pre-packed chicken meat on a daily basis (breast and thigh). The estimated daily carnosine intake was 410 mg for the control group and 590 mg for the CAR group. Study protocol required two visits to the Laboratory for Clinical and Sports Physiology (Department of Physiology and Immunology, Faculty of Medicine, University of Osijek, Osijek, Croatia) during which all measurements were performed and samples were collected. Figure 1 shows the study design.

Study participants were randomized, and a unique code was created for each participant. Labels consisted of letters indicating the treatment and participant characteristics, followed by the participant number and measurement identifier. Participants recorded their diet using a validated food frequency questionnaire and were regularly contacted by email/phone to ensure protocol compliance. Throughout the study, they did not consume any additional carnosine supplements. All participants provided signed informed consent and were briefed on the study protocols and procedures. This study was approved by the Ethics Committee of the Faculty of Medicine Osijek (Cl: 602-04/23-08/03; No: 2158-61-46-23-89) on 24 May 2023. A partner research group from the Faculty of Agrobiotechnical Sciences Osijek, University of Osijek, Croatia fed Ross 308 broilers with either a standard diet or one enriched with β-alanine, L-histidine, and MgO to produce carnosine-enriched chicken meat. Analysis showed higher carnosine levels in enriched diet fed chickens (846.8 mg/kg in breast and 341.5 mg/kg in thigh) compared to controls (537.8 mg/kg and 167.7 mg/kg, respectively). The full study protocol, including a CONSORT diagram and enriched meat production, has been previously published in Perić et al. (2025) [10]. Hereby, only a brief summary is provided as the results presented in this paper refer to the same study participants as in said paper.

### 2.2. Sampling and Anthropometric Measurements

Venous blood samples were collected after an overnight fasting on the two visits to the laboratory. Analyses included total blood count (TBC), transferrin, fasting glucose, and high-sensitivity C-reactive protein (hsCRP). Analysis was performed at the Department of Clinical Laboratory Diagnostics, Osijek University Hospital, Osijek, Croatia. Serum was isolated from whole blood samples by centrifugation at 3600 RPMI for 10 min (Rotina 380, Hettich GmbH & Co. KG, Tuttlingen, Germany). The serum was then collected, aliquoted, and stored at −80 °C freezer until further analysis. Peripheral blood mononuclear cells (PBMC) were isolated using Ficoll-Paque^®^ PLUS media (GE Healthcare Bio-Sciences AB, Uppsala, Sweden) for density gradient centrifugation as described in a previously published paper by our research team [32]. Cells were counted, aliquoted correctly, and stored in the freezer until further proceedings.

Body mass index (BMI) was calculated using the standard formula (kg/m^2^) based on body mass and height measured at each visit by the researcher. Waist-to-hip ratio (WHR) was calculated from the measurements obtained by the researcher with a tape measure.

### 2.3. Antioxidative Enzyme Activity

SOD, GPx, and CAT enzyme activity assays were conducted on undiluted serum samples using a Spark multimode microplate reader and SparkControl software v2.1 (Tecan, Männedorf, Switzerland) at the Subdepartment for Biochemistry and Molecular Biology, Department of Biology, J. J. Strossmayer University in Osijek. Assays followed established protocols with volume and extinction factor adjustments for microplate format, as described previously [20,33].

CAT activity was measured using 50 mM phosphate buffer and 0.036% H_2_O_2_ as substrate, based on Aebi’s method [34]. Absorbance decrease at 240 nm was recorded every 10 s over 2 min. One unit of CAT activity corresponds to the decomposition of 1 μmol H_2_O_2_ per minute. SOD activity was assessed by monitoring the inhibition of cytochrome C reduction at 550 nm in a xanthine–xanthine oxidase system, following the modified protocol of Flohé and Otting [35]. One unit of SOD was defined as the amount causing 50% inhibition of cytochrome C reduction. Glutathione peroxidase (GPx) activity was determined by the rate of NADPH oxidation to NADP^+^, measured as a decrease in absorbance at 340 nm over 5 min, according to Wendel’s method [36]. One unit of GPx activity corresponds to the oxidation of 1 μmol of reduced glutathione per minute under standard conditions (pH 7.0, 25 °C).

All enzyme activities were normalized to total protein content and expressed as U/mg protein. Protein concentration in serum was measured using the Bradford assay (Bradford Reagent B6916, Sigma Aldrich, St. Gallen, Switzerland) at 595 nm, with bovine serum albumin as standard.

### 2.4. Flow Cytometry

Reactive oxygen species (ROS) production in PBMCs was evaluated using flow cytometry (FACS Canto II, BD Biosciences (Bistra, Croatia); 488 nm excitation, 530/30 bandpass filter) and analyzed with Flow Logic V8 software (Inivai Technologies, Mentone, Australia), following established protocols [37,38]. To assess specific ROS production in PBMCs, DCF-DA was used to detect hydrogen peroxide (H_2_O_2_) and peroxynitrite (ONOO^−^), while DHE was applied for superoxide anion (O_2_^−^). ROS production was stimulated by adding phorbol 12-myristate 13-acetate (PMA) to all samples. Signals were collected in the FLH-1 and FLH-2 channels and the geometric mean fluorescence intensity (GeoMean) of the gated positive population on the histogram was used for data analysis. Data are presented as geometric mean fluorescence intensity (GMFI) in respective channels (FLH-1 for DCF-DA and FLH-2 for DHE).

### 2.5. Enzyme-Linked Immunosorbent Assay (ELISA)

Serum concentration of 8-iso Prostaglandin F2α (8-iso PGF 2α) was measured by a commercially available enzyme-linked immunosorbent assay (ELISA) kit (MyBioSource, MyBioSource Inc., San Diego, CA, USA) on a compact absorbance reader for 96-well microplates (BioRad PR 3100 TSC, Bio-Rad Laboratories, Hercules, CA, USA) at 450 nm.

### 2.6. Luminex Assay

Concentrations of cell adhesion molecules (CAMs): intercellular adhesion molecule 1 (ICAM-1), vascular cell adhesion molecule 1 (VCAM-1), E-selectin, and endoglin—were measured in serum samples before and after the dietary intervention. Quantification was performed using Invitrogen ProcartaPlex magnetic bead-based kits (Thermo Fisher Scientific, Waltham, MA, USA), on a Luminex 200 platform (Luminex Corp., Austin, TX, USA). Analyses were conducted at the Laboratory of Molecular and HLA Diagnostics, University Hospital Osijek (Osijek, Croatia). Data were processed using ProcartaPlex Analyst software (eBioscience, Thermo Fisher Scientific, Waltham, MA, USA) and reported as concentrations in picograms per millilitre (pg/mL).

### 2.7. Statistical Analysis

All statistical analyses and visualization were performed in Microsoft Excel 2021 (Microsoft^®^ Excel^®^ LTSC MSO 64-bit, Microsoft Corporation, Redmond, WA, USA), Graph Pad Prism v8 (GraphPad Software, San Diego, CA, USA), and SigmaPlot v11.2 (Systat Software, Inc., Chicago, IL, USA). Results were expressed as the arithmetic mean and standard deviation (SD). Preliminary study was performed to obtain the data required for sample size calculations. It was determined that the sample size for the current study is 15 participants per group for a statistical strength of 80% with statistical significance of 0.05 for ANOVA. The normality of distributions was tested by the Shapiro–Wilk test. Student’s *t*-test and Mann–Whitney tests were used for group comparisons, while Paired *t*-test and Wilcoxon rank-sum tests were used to test the differences between the measurements within a group. A One-Way ANCOVA was used to compare group means post-intervention while adjusting for baseline values (pre-intervention) as a covariate. A two-way ANOVA with repeated measures was performed to test the difference between the means of the measurements at each time point between the groups and within the groups, and to test whether the difference between the interventions depended on the time at which the measurement was performed. Two-tailed *p* < 0.05 was considered significant.

## 3. Results

### 3.1. General Characteristics, Anthropometric and Biochemical Parameters of Study Participants

General characteristics of study participants alongside anthropometric and biochemical parameters are shown in Table 1. Participants in both groups were closely the same age, with normal BMI (according to the WHO criteria for European population weight classification 18.5–24.9 kg/m^2^) and WHR with no significant differences between the CTRL and CAR groups, neither before nor after the dietary protocol within respective study groups. Slight but significant differences that were observed within experimental groups in leukocyte count (*p* = 0.011), haemoglobin (*p* = 0.03), and haematocrit (*p* = 0.035) were all within the reference range for the general population. There were no significant changes in TBC, fasting glucose, and serum hsCRP following dietary protocol in either the CTRL or CAR group.

### 3.2. CAT, GPx, and SOD Serum Activity

**Figure 2:**
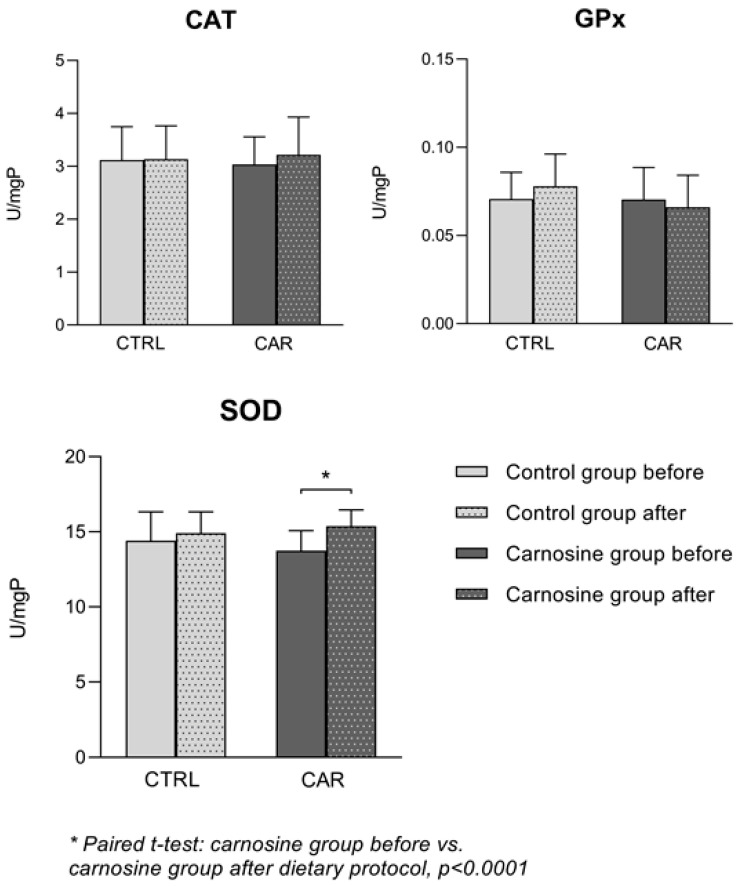
Enzyme antioxidant activity in serum samples of competitive athletes following regular (control group) or carnosine-enriched chicken meat (carnosine group) consumption. CTRL—control group; CAR—carnosine group; SOD—superoxide dismutase; GPx—glutathione peroxidase; CAT—catalase; U/mgP—units per milligram of protein. Paired *t*-test or Wilcoxon signed-rank test (pre vs. post within groups); One-Way ANCOVA (post-control vs. post-carnosine adjusted for baseline values); * *p* < 0.05.

Activities of antioxidant enzymes in serum samples of competitive athletes before and after the dietary protocols are shown in Figure 2. There were no significant changes in CAT and GPx serum activity in the CTRL or CAR group following the dietary protocols compared to baseline. There was a significant increase in SOD activity following consumption of carnosine-enriched chicken compared to baseline values (*p* < 0.0001).

### 3.3. Hydrogen Peroxide, Peroxynitrite, and Superoxide Anion Intracellular Production in PBMCs

**Figure 3:**
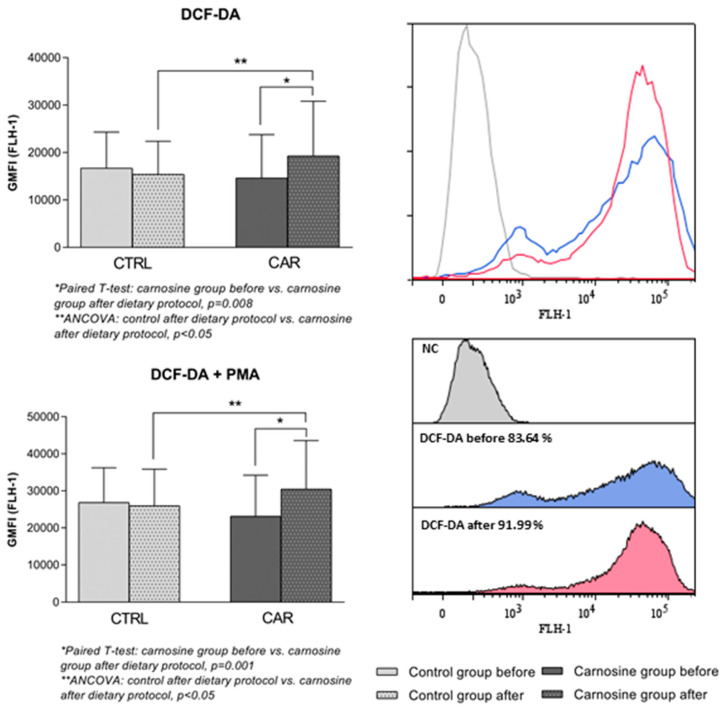
Intracellular production of hydrogen peroxide and peroxynitrite in PBMCs of competitive athletes following regular (control group) or carnosine-enriched chicken meat (carnosine group) consumption with representative histograms. CTRL—control group; CAR—carnosine group; GMFI—geometric mean fluorescence intensity; DCF-DA—2′,7′-dichlorodihydrofluorescein diacetate; PMA—Phorbol 12-myristate 13-acetate; NC—negative control. Paired *t*-test or Wilcoxon signed-rank test (* pre vs. post within groups); One-Way ANCOVA (** post-control vs. post-carnosine adjusted for baseline values); *p* < 0.05.

**Figure 4:**
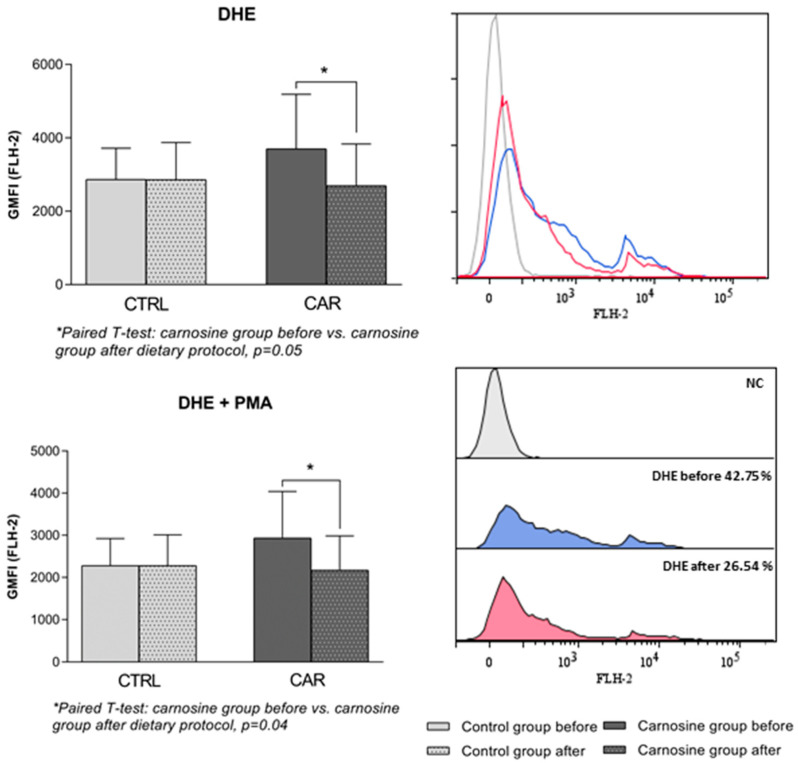
Intracellular production of superoxide in PBMCs of competitive athletes following regular (control group) or carnosine-enriched chicken meat (carnosine group) consumption with representative histograms. CTRL—control group; CAR—carnosine group; GMFI—geometric mean fluorescence intensity; DHE—Dihydroethidium; PMA—Phorbol 12-myristate 13-acetate; NC—negative control. Paired *t*-test or Wilcoxon signed-rank test (pre vs. post within groups); One-Way ANCOVA (post-control vs. post-carnosine adjusted for baseline values); * *p* < 0.05.

Intracellular ROS production in PBMCs of competitive athletes following the dietary protocol is shown in Figure 3 and Figure 4. There was a significant increase in hydrogen peroxide and peroxynitrite levels (*p* = 0.008), while a significant decrease in superoxide anion was observed (*p* = 0.05), in the CAR group following the dietary protocol compared to baseline. A significant interaction between time and treatment was observed in hydrogen peroxide and peroxynitrite intracellular production (*p* = 0.012), indicating that the change over time depends on the group. There were no significant differences in ROS production in the CTRL group following the dietary protocol compared to baseline.

### 3.4. Serum Concentration of 8-iso Prostaglandin F2α (8-iso PGF 2α)

**Figure 5:**
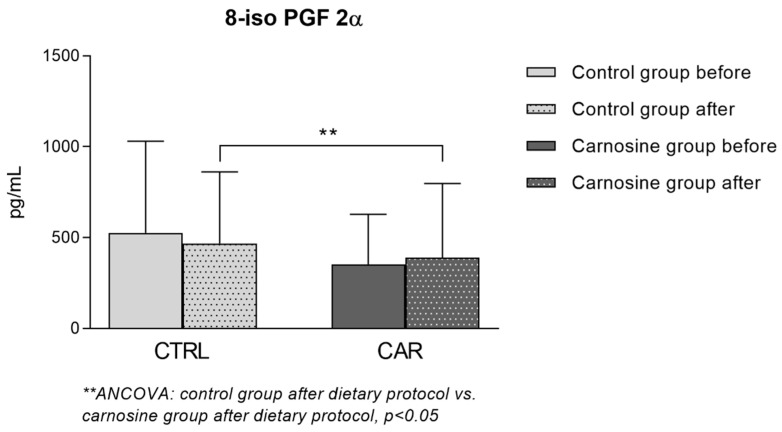
8-iso prostaglandin F2α (8-iso PGF 2α) concentration in serum samples of competitive athletes following regular (control group) or carnosine-enriched chicken meat (carnosine group) consumption. CTRL—control group; CAR—carnosine group. One-Way ANCOVA (** post-control vs. post-carnosine adjusted for baseline values); *p* < 0.05.

Serum concentration of 8-iso PGF 2α in CTRL and CAR groups is shown in Figure 5. A significant interaction between time and treatment (*p* < 0.0001) was observed. Consumption of control or carnosine-enriched chicken for three weeks did not significantly change the serum concentrations of 8-iso PGF 2α compared to baseline. However, when adjusted for baseline values, there was a significant difference between the CTRL and CAR groups after the dietary protocol (*p* = 0.025). The CAR group had a significantly lower concentration of 8-iso PGF 2α following the dietary protocol when compared to the CTRL group values.

### 3.5. Serum Cell Adhesion Molecules Concentration

**Figure 6:**
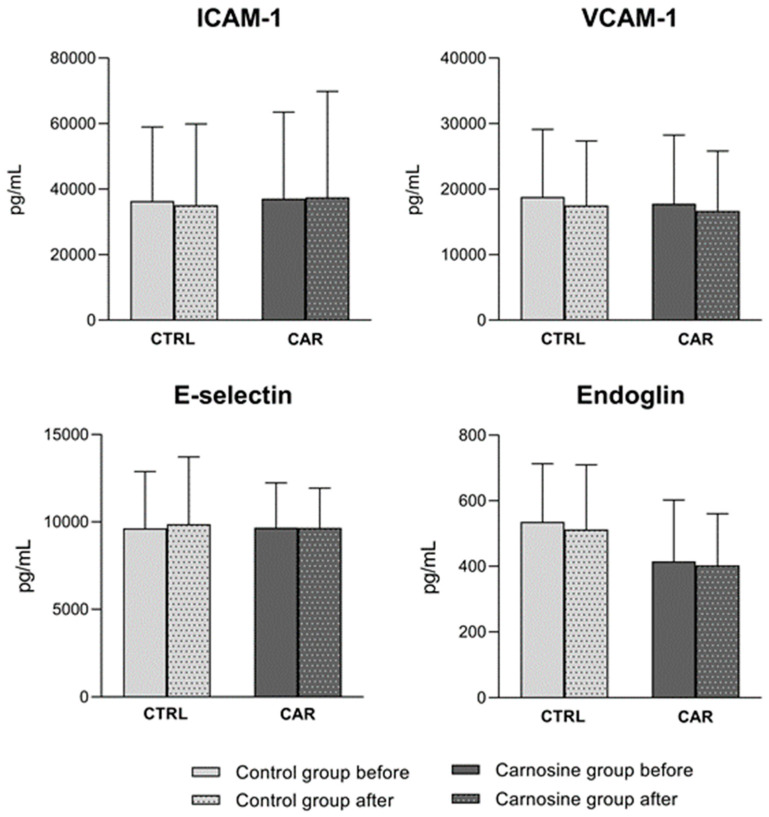
Cell adhesion molecule concentrations in serum samples of competitive athletes following regular (control group) or carnosine-enriched chicken meat (carnosine group) consumption. CTRL—control group; CAR—carnosine group; ICAM-1—Intercellular Adhesion Molecule 1; VCAM-1—Vascular Cell Adhesion Molecule-1. Paired *t*-test or Wilcoxon signed-rank test (pre vs. post within groups); One-Way ANCOVA (post-control vs. post-carnosine adjusted for baseline values); *p* < 0.05.

CAM serum concentrations are shown in Figure 6. There were no significant changes in concentrations of ICAM-1, VCAM-1, E-selectin, and Endoglin in either the CTRL or CAR group after the respective dietary protocols compared to baseline. Furthermore, no significant differences were observed between CTRL and CAR groups post-protocol values when adjusted for baseline values. Even though a significant interaction between time and treatment was observed in all measured CAM concentrations (*p* < 0.0001; *p* = 0.005; *p* < 0.0001; *p* < 0.0001), suggesting that CAMs exhibited different patterns of change over time depending on the intervention, these changes were not pronounced enough to reach statistical significance within groups or in the final between-group comparison.

## 4. Discussion

The current lack of randomized clinical studies, particularly related to the use of dietary supplements and functional foods, highlights the need for greater attention from both the public and the scientific community. It is essential to elucidate the mechanisms of action and effects of individual compounds on well-being, as well as to explore their potential in preventive care. This study contributes valuable clinical data by demonstrating the antioxidative effects in the endothelium of carnosine supplementation in the form of functional food in humans. These are key findings of the present study: (a) SOD enzyme activity was significantly increased in response to consumption of carnosine-enriched chicken meat compared to baseline; (c) in the group that consumed carnosine-enriched chicken meat, intracellular superoxide anion was lowered, while intracellular hydrogen peroxide and peroxynitrite were elevated compared to baseline; (b) lipid peroxidation was lower in athletes consuming carnosine-enriched chicken meat compared to controls following dietary protocols.

Antioxidant properties of carnosine have been excessively studied and verified through a number of studies conducted over the last 30 years. Kang et al. (2002) found that carnosine and related compounds (homocarnosine and anserine) inhibit oxidation in Cu/Zn-SOD induced by 2,2′-azobis(2-amidinopropane) dihydrochloride (AAPH) radicals in vitro [39]. This was corroborated by Stvolonskii et al. (2002) using in vivo models, supporting the protective and antioxidative effects of carnosine under two different models for oxidative stress [40]. Inhibition of lipid peroxidation by carnosine has also been studied in the food industry citing this dipeptide as an excellent potential natural antioxidant in processed food [41,42]. Fresta et al. (2017) found that macrophages may increase carnosine uptake as a defence mechanism during inflammation (LPS and IFNγ stimulation) and under oxidative stress [43]. Our results are in agreement with these findings, as the serum concentration of 8-iso PGF 2α, a marker for lipid peroxidation, was significantly lower in the CAR group compared to the CTRL group when adjusted for baseline values.

Further, carnosine’s activities as a ROS scavenger and a copper chelator appear to protect SOD from oxidative stress. This was previously reported by several studies conducted on rodent models, were carnosine treatment effectively increased SOD activity/expression in different tissues and blood serum [20,21,44]. Similar results were reported in vitro where carnosine increased antioxidative enzymes expression (SOD, GPx) in macrophages [45], human lymphocytes [46], and HK-2 cells [16]. Our data go in line with these findings, showing increased SOD serum activity and significantly decreased superoxide anion intracellular production in PBMCs of athletes following carnosine-enriched chicken meat consumption. SOD catalyzes the degradation of superoxide anion to hydrogen peroxide [47,48], which also explains the observed increase in peroxide production in the carnosine group following the dietary protocol. Hydrogen peroxide is far less reactive and more stable than the superoxide anion, which contains an unpaired electron, making it highly susceptible to oxidation-reduction reactions [49]. Additionally, it is known that extensive exercise increases lipid peroxidation and overall oxidative stress [50,51,52].

The present study showed that carnosine supplementation in healthy athletes did not induce significant changes in CAMs or endoglin concentrations in either experimental group. Earlier studies reported that carnosine inhibits cancer cell adhesion, migration, invasion, and proliferation in human colorectal cancer (HCT-116) (integrin β1) and ovarian cancer cells, while it also reduces ICAM-1 and E-selectin levels in umbilical vein cells (EA.hy926) [53,54]. Wu et al. (2019) also reported that carnosine inhibited the NF-κB pathway by reducing IκB phosphorylation in the EA.hy926 cell line [54]. This is a promising perspective for future research since there is a suggested strong effect of carnosine supplementation on adhesion, especially in carcinogenic cell lines. The result that carnosine supplementation in this study did not induce significant changes in the concentrations of biomarkers of endothelial activation could be a consequence of the limited duration of the intervention or the initial optimal endothelium status of healthy athletes.

The strengths of this study include its randomized controlled trial design, which minimizes bias and strengthens the validity of the findings, as well as the use of a homogeneous and well-defined population of young, healthy male athletes, thereby reducing heterogeneity and potential confounding effects. Importantly, the intervention was implemented by functional food—carnosine-enriched chicken meat—rather than supplementation, offering a translational and practical approach with high applicability to everyday nutrition. A comprehensive set of oxidative stress and antioxidant defence biomarkers was assessed using validated and sensitive laboratory methods, ensuring a multidimensional evaluation of redox status. The specific focus of this study on competitive athletes makes the findings particularly relevant for populations with elevated oxidative stress due to intense physical activity.

Limitation of this study was the inability to directly assess carnosine levels in serum/plasma or muscle tissue after supplementation, given the rapid breakdown of circulating carnosine in human blood and the invasive procedure required for muscle biopsy. This limitation was mitigated through regular communication with participants to ensure adherence to the prescribed dietary protocol.

## 5. Conclusions

The practical implications of this study highlight the potential of functional food strategies, such as carnosine-enriched chicken meat, to serve as a feasible and accessible nutritional approach for enhancing antioxidant defence and mitigating exercise-induced oxidative stress in athletes. Unlike supplementation, functional foods can be easily incorporated into regular dietary habits, increasing compliance and long-term applicability. By demonstrating improvements in enzymatic antioxidant activity and reductions in oxidative damage markers, this intervention may contribute to better recovery, reduced cellular damage, and long-term vascular health in populations exposed to high oxidative stress, such as competitive athletes. These findings also provide a translational framework for extending the use of carnosine-enriched foods to broader populations at risk of oxidative stress–related conditions, thereby supporting preventive health strategies.

To conclude, this study highlights the promising antioxidative and vascular/hemo benefits of carnosine supplementation through functional food in humans, through a significant increase in SOD activity, decrease in intracellular superoxide production, and overall lower serum lipid peroxidation following consumption of enriched chicken in competitive athletes. Given the limited number of randomized clinical trials in this area, further research is warranted to elucidate the underlying mechanisms.

## Figures and Tables

**Table 1:** General characteristics, anthropometric and biochemical parameters of study participants.

Parameter	CTRL			CAR			*p* ^‡^
N	16			19			
Age (years)	22.6 (5.9)			22.3 (4.8)			0.072
	Before	After	*p* ^†^	Before	After	*p* ^†^	*p* ^‡^
BMI (kg/m^2^)	24.5 (2.5)	24.4 (2.5)	0.259	24.2 (2.0)	24.2 (1.9)	0.871	0.366
WHR	0.82 (0.05)	0.81 (0.04)	0.382	0.82 (0.04)	0.82 (0.04)	0.813	0.92
Erythrocytes (×10^12^/L)	5.2 (0.3)	5.1 (0.3)	0.065	5.0 (0.3)	5.0 (0.4)	0.117	0.411
Haemoglobin (g/L)	150.4 (6.2)	146.8 (6.9)	0.03 *	144.1 (7.3)	144.4 (10.9)	0.229	0.561
Haematocrit (%)	0.454 (0.017)	0.445 (0.023)	0.035 *	0.434 (0.021)	0.440 (0.030)	0.034 *	0.425
Leukocytes (×10^9^/L)	6.6 (1.0)	5.9 (1.1)	0.011 *	6.3 (1.6)	5.8 (1.3)	0.155	0.716
Thrombocytes (×10^9^/L)	241.7 (36.2)	239.2 (36.3)	0.729	227.6 (39.9)	229.9 (30.2)	0.726	0.75
Transferrin (g/L)	2.8 (0.3)	2.7 (0.4)	0.211	2.8 (0.4)	2.8 (0.4)	0.712	0.448
Glucose (mmol/L)	4.7 (0.4)	4.8 (0.4)	1	4.8 (0.4)	4.8 (0.5)	0.951	0.786
hsCRP (mg/L)	2.6 (7.5)	1.6 (3.7)	0.359	1.1 (1.0)	1.5 (1.9)	0.839	0.97

Data are presented as mean ± standard deviation (SD). N—number of participants; CTRL—control group; CAR—carnosine group; BMI—body mass index; hsCRP—high-sensitivity C-reactive protein. ^†^ Paired-samples *t*-test or Wilcoxon signed-rank test (before vs. after within groups); ^‡^ One-Way ANCOVA (post-control vs. post-carnosine adjusted for pre-values); * *p* < 0.05.

## Data Availability

The original contributions presented in this study are included in the article. Further inquiries can be directed to the corresponding authors.
